# The impact of tumor characteristics on cardiovascular disease death in breast cancer patients with CT or RT: a population-based study

**DOI:** 10.3389/fcvm.2023.1149633

**Published:** 2023-05-09

**Authors:** Kaiyi Chi, Zehao Luo, Hongjun Zhao, Yemin Li, Yinglan Liang, Zhaoling Xiao, Yiru He, Hanbin Zhang, Zaiying Ma, Liangjia Zeng, Ruoyun Zhou, Manting Feng, Wangen Li, Huying Rao, Min Yi

**Affiliations:** ^1^ Department of Endocrinology, The Second Affiliated Hospital of Guangzhou Medical University, Guangzhou, China; ^2^ Department of Clinical Medicine, The Second Clinical College of Guangzhou Medical University, Guangzhou, China; ^3^ Cardiovascular Medicine and Cardio-Oncology Group, Medical Exploration and Translation Team, Guangzhou, China; ^4^ Department of Clinical Medicine, The Sixth Clinical College of Guangzhou Medical University, Guangzhou, China; ^5^ Department of Clinical Medicine, The First Clinical College of Guangzhou Medical University, Guangzhou, China; ^6^ Department of Anesthesiology, The Second Clinical College of Guangzhou Medical University, Guangzhou, China; ^7^ Department of Clinical Medicine, The Third Clinical College of Guangzhou Medical University, Guangzhou, China; ^8^ Department of Radiological Oncology, Nanfang Hospital, Southern Medical University, Guangzhou, China

**Keywords:** breast cancer, cardio-oncology, tumor size, tumor stage, cardiovascular diseases death, SEER, chemotherapy, radiotherapy

## Abstract

**Background:**

Previous studies focused on the impact of cardiovascular diseases (CVD) risk factors in breast cancer patients with chemotherapy (CT) or radiotherapy (RT). This study aimed to identify the impact of tumor characteristics on CVD death in these patients.

**Methods:**

Data of female breast cancer patients with CT or RT between 2004 and 2016 were included. The risk factors of CVD death were identified using Cox regression analyses. A nomogram was constructed to evaluate the predicted value of tumor characteristics, and then validated by the concordance indexes (C-index) and calibration curves.

**Result:**

A total of 28,539 patients were included with an average follow-up of 6.1 years. Tumor size > 45 mm (adjusted HR = 1.431, 95% CI = 1.116–1.836, *P* = 0.005), regional (adjusted HR = 1.278, 95% CI = 1.048–1.560, *P* = 0.015) and distant stage (adjusted HR = 2.240, 95% CI = 1.444–3.474, *P* < 0.001) were risk factors of CVD death for breast cancer patients with CT or RT. The prediction nomogram of tumor characteristics (tumor size and stage) on CVD survival was established. The C-index of internal and external validation were 0.780 (95% Cl = 0.751–0.809), and 0.809 (95% Cl = 0.768–0.850), respectively. The calibration curves showed consistency between the actual observation and nomogram. The risk stratification was also significant distinction (*P* < 0.05).

**Conclusion:**

Tumor size and stage were related to the risk of CVD death for breast cancer patients with CT or RT. The management of CVD death risk in breast cancer patients with CT or RT should focus not only on CVD risk factors but also on tumor size and stage.

## 1. Introduction

Breast cancer is the most prevalent cancer among females in the United States (1). Approximately one women in eight can be diagnosed with breast cancer (2). The patient survival rate is greatly improved with the development of cancer treatment technology. It was estimated that the death rate of breast cancer survivors dropped by 43% in total during 1989–2020 (2). Nevertheless, cardiovascular diseases (CVD) events increase more and more concerns among these survivors, especially for those who received chemotherapy (CT) or radiotherapy (RT). It was suggested that CVD surpasses the risk of cancer and becomes the leading cause of death among breast cancer survivors (3). In addition, prior studies mostly supported that the cumulative cardio-toxicity of CT or RT increases the risk of CVD death in breast cancer patients (4–8). Since those who accepted CT or RT are at high risk of CVD death, we focused on these patients and selected them as our study population and then identified risk factors of CVD death in breast cancer patients to control the hazards of CVD and improve these patients' outcomes.

Most previous studies focused on anticancer treatment and traditional CVD risk factors, including diabetes, atrial fibrillation, and heart failure hypertension in breast cancer patients with CT or RT, and the impact of tumor characteristics on breast cancer survivors was neglected (9–12). Increasing studies showed that breast cancer itself contributes to the risk of CVD death (13–17), suggesting that the association between tumor characteristics and CVD death risk needs further researches. Tumor characteristics, including tumor size, stage, grade, estrogen receptor (ER), progesterone receptor (PR) and human epidermal receptor 2 (HER2) status, are closely related to breast cancer survivors' overall survival (18–21). However, whether these risk factors increase the risk of CVD death remains unclear. In order to solve this problem, further studies should comprehensively analyze the impact of tumor characteristics on the risk of CVD death in breast cancer patients with CT or RT.

We conducted a population-based study based on the Surveillance, Epidemiology and End Results (SEER) database to define the risk of CVD death in breast cancer patients with CT or RT. Tumor characteristics were systematically considered to clarify the risk factors of CVD from a new perspective. Nomogram was utilized to quantify and visualize the risk of CVD death among each factor. Our study may provide evidence to monitor the risk of CVD death regularly and implement personalized precision treatment in breast cancer patients with CT or RT.

## 2. Methods

### 2.1. Study population and design

The female breast cancer patients with CT or RT from 2004 to 2016 in the SEER database were extracted and screened. The inclusion criteria were defined as follows: (1) case selection (site and morphology, primary site-labeled) = “C50.x”; (2) participants with only a single primary tumor; (3) pathological diagnosis between 2004 and 2016; (4) participants with active follow-up; (5) participants who received CT or RT. The exclusion criteria were defined as follows: (1) male patients; (2) unknown causes of deaths; (3) unknown surgery; (4) unknown stage; (5) unknown ER status; (6) unknown PR status; (6) unknown race; (7) unknown tumor size; (8) unknown grade; (9) unknown laterality.

### 2.2. Participant variables and outcomes

Participant variables included year of diagnosis (2004–2007, 2008–2011, 2012–2016), age of diagnosis (≤ 65 years,>65 years), race (white, black, other), marital status (married, unmarried), laterality (right, left), histologic subtypes (ductal, lobular, mixed, other), tumor size (≤ 45 mm,>45 mm), grade (low, high), stage (localized, regional, distant), ER status (negative, positive), PR status (negative, positive), HER2 status (negative, positive, unknown), and surgery (no evidence, yes). The size of tumor was stratified by using the X-tile program (Yale University, New Haven, Connecticut, USA) (22) (Supplementary Material Figure S1). The histopathology was classified based on International Classification of Diseases for Oncology, 3rd edition (ICD-O-3) codes and detail information was showed in the Supplementary method. We identified 47 mm as the optimal cut-off value of tumor size and rounded it to 45 mm. Therefore, according to tumor size, this cohort was divided into two groups, including ≤45 mm and >45 mm.

In our study, the causes of death were classified as CVD and non-CVD. According to the International Classification of Diseases-10 (ICD-10) codes, the CVD death was defined as death from heart disease (I00-I09, I11, I13, I20-I51), hypertension without heart disease (I10, I12), cerebrovascular disease (I60-I69), atherosclerosis (I70), aortic aneurysm and dissection (I71) and other arterial, arteriolar and capillary diseases (I72-I78) (23). The follow-up time was calculated as the period from the first breast cancer diagnosis to death or the last follow-up. The last follow-up date was on December 31, 2016.

### 2.3. Statistical analysis

Univariate and multivariate Cox regression analyses (enter method) were used to identify the risk factors of CVD death in breast cancer patients with CT or RT (24–26). A sensitivity analyze (3 models with increasing degrees of adjustment) was conducted to adjust for potential confounding variables at baseline (27, 28). In detail, model 1 was adjusted for all variables with *P*-values less than 0.05 at univariate analysis, including age at diagnosis, marital status, race, tumor size, and stage. Model 2 was the same as model 1, and further included other tumor characteristics including grade, laterality, histologic subtypes, ER status, HER2 status, and PR status. Model 3 was adjusted for all variables in the baseline.

Participants were randomly divided into a training cohort and a validation cohort at a ratio of 7:3 (29). Categorical variables in baseline characteristics were compared by the chi-square test. In the training cohort, the univariate Cox regression analysis was used for preliminary screening, while prognostic variables with statistical differences were further evaluated in the multivariate Cox regression. A nomogram for 5-, 8-, and 10-year CVD survival was established according to the results of the multivariate Cox regression analysis for the training cohort.

The concordance indexes (C-index) and calibration curves were used to analyze and assess the accuracy of the nomogram (29). The value of the C-index varies from 0.5 to 1.0, with 0.5 indicating random chance and 1.0 indicating great consistency between the training cohort and validation cohort. When a C-index value is 0.7 or higher, two cohorts are considered to have a good consistency. The calibration curve was plotted to evaluate the predicted and observed survival curves. The closer the predicted curve is to the actual curve, the more accurate the model is. Based on the nomogram score of each patient, we used X-tile to divide patients into three groups: low-risk (0–5 points), intermediate-risk (5.1–12.4 points), and high-risk (12.5–20.8 points) (Supplementary Material Figure S2).

SPSS version 25.0 (SPSS, Chicago, IL) was utilized to conduct chi-square test and perform the univariate and multivariate Cox regression. R software version 3.4.4 (https://www.r-project.org) was used to develop and verify the nomogram. A *P* value < 0.05 was indicated statistically significant.

## 3. Result

### 3.1. Baseline characteristics

A total of 28,539 female breast cancer patients with CT or RT between 2004 and 2016 were included, with an average follow-up of 6.1 years (SD 0.1 years). Among the 28,539 participants included, 77.7% were aged at diagnosis ≤65 years, 78.4% were white, 62.1% were married, 51.1% were left tumor and 75.1% of histologic subtypes were ductal. The proportion of tumor size ≤ 45 mm (81.9%) was higher than that of tumor size > 45 mm (18.1%). For the year of diagnosis, 29.1% were diagnosed in 2004–2007, 31.8% in 2008–2011 and 39.1% in 2012–2016. For the grade and stage, 57.4% were high grade, 43.3% were localized stage, 51.3% were regional stage and 5.4% were distant stage. The proportion of positive ER status were 78.4%, the proportion of positive PR status were 67.6% and the proportion of positive HER2 status were 12.1%. A high percentage had surgery (97.3%) (Table 1).

**Table 1:** Baseline characteristics of 28,539 breast cancer patients with CT or RT.

	Patients (*N* = 28,539)	
	Number	Proportion, %
Age at diagnosis		
≤65	22,166	77.7
>65	6,373	22.3
Race		
White	22,376	78.4
Black	3,543	12.4
Other*	2,620	9.2
Marital status		
Married	17,724	62.1
Unmarried	10,815	37.9
Laterality		
Right	13,948	48.9
Left	14,591	51.1
Histologic subtypes		
Ductal	21,447	75.1
Lobular	2,519	8.8
Mixed	3,464	12.1
Other	1,109	3.9
Tumor size		
≤45 mm	23,361	81.9
>45 mm	5,178	18.1
Year of diagnosis		
2004–2007	8,301	29.1
2008–2011	9,075	31.8
2012–2016	11,163	39.1
Grade		
Low	12,165	42.6
High	16,374	57.4
Stage		
Localized	12,354	43.3
Regional	14,637	51.3
Distant	1,548	5.4
ER status		
Negative	6,160	21.6
Positive	22,379	78.4
PR status		
Negative	9,249	32.4
Positive	19,290	67.6
HER2 status		
Negative	11,706	41.0
Positive	3,457	12.1
Unknown	13,376	46.9
Surgery		
No evidence	771	2.7
Yes	27,768	97.3

^*^ Other includes American Indian/Alaska Native and Asian/Pacific Islander.

CT, chemotherapy; RT, radiotherapy; ER, estrogen receptor; PR, progesterone receptor; HER2, human epidermal receptor 2.

### 3.2. Risk factors for CVD death in breast cancer patients With CT or RT

Univariate Cox regression analysis showed that tumor size, stage, age at diagnosis, marital status and race were related to CVD death risk for breast cancer patients with CT or RT (all *P* < 0.001). Tumor laterality, histologic subtypes, year of diagnosis, tumor grade, positive ER status, positive PR status, positive HER2 status and surgery were not significantly associated with CVD death in those patients (all *P* > 0.05) (Supplementary Material Table S1).

Particularly, tumor size > 45 mm (crude HR = 1.551, 95% CI = 1.229–1.957, *P* < 0.001) and distant stage (crude HR = 2.391, 95% CI = 1.572–3.636, *P* < 0.001) were correlated with higher CVD death risk among breast cancer patients with CT or RT (Table 2). To adjust for confounding factors, sensitivity analysis was performed to further identify the impact of tumor characteristics (tumor size and stage) on CVD death. According to adjustment in model 1, robust adjusted hazard ratios (HRs) were shown on tumor size >45 mm (adjusted HR = 1.431, 95% CI = 1.116–1.836, *P* = 0.005), regional (adjusted HR = 1.278, 95% CI = 1.048–1.560, *P* = 0.015) and distant stage (adjusted HR = 2.240, 95% CI = 1.444–3.474, *P* < 0.001). In further adjustment for other tumor characteristics in model 2, adjusted HR of tumor size > 45 mm decreased to 1.427-fold, compared to the patients with tumor size ≤ 45 mm (adjusted HR = 1.427, 95% CI: 1.110–1.834, *P* = 0.006); adjusted HR of distant stage decreased to 2.170-fold and regional stage decreased to 1.235-fold, compared to the patients with localized stage(adjusted HR = 2.170, 95% CI = 1.395–3.375, *P* = 0.001; adjusted HR = 1.235, 95% CI = 1.009–1.511, *P* = 0.040). After further adjustment for all variables at baseline in model 3, adjusted HR of tumor size and stage remained stable (adjusted HR for tumor size > 45 mm = 1.424, 95% CI = 1.108–1.831, *P* = 0.006; adjusted HR for regional stage = 1.231, 95% CI = 1.006–1.507, *P* = 0.043; adjusted HR for distant stage = 2.071, 95% CI = 1.306–3.285, *P* = 0.002) (Table 2).

**Table 2:** Cox regression analysis of the impact of tumor characteristics on the risk of CVD death.

Variable	Crude HR		Model 1^a^		Model 2^b^		Model 3^c^	
	HR (95% CI)	*P* Value	HR (95% CI)	*P* Value	HR (95% CI)	*P* Value	HR (95% CI)	*P* Value
Tumor size								
≤45 mm	Reference		Reference		Reference		Reference	
>45 mm	1.551 (1.229–1.957)	< 0.001	1.431 (1.116–1.836)	0.005	1.427 (1.110–1.834)	0.006	1.424 (1.108–1.831)	0.006
Stage								
Localized	Reference		Reference		Reference		Reference	
Regional	1.143 (0.945–1.383)	0.168	1.278 (1.048–1.560)	0.015	1.235 (1.009–1.511)	0.040	1.231 (1.006–1.507)	0.043
Distant	2.391 (1.572–3.636)	< 0.001	2.240 (1.444–3.474)	< 0.001	2.170 (1.395–3.375)	0.001	2.071 (1.306–3.285)	0.002

^a^ Model 1: HRs were adjusted for statistically significant factors according to univariate analysis (age at diagnosis, marital status, race and tumor size, and stage).

^b^ Model 2: It is the same as Model 1, and also includes other tumor characteristics including, grade, laterality, histologic subtypes, estrogen receptor, human epidermal receptor 2, and progesterone receptor.

^c^ Model 3: HRs were adjusted for all variables in the baseline.

CVD, cardiovascular diseases; HR, hazard ratio; CI, confidence interval.

### 3.3. The prediction nomogram of tumor characteristics (tumor size and stage) on CVD survival

The training cohort included 19,977 patients and the validation cohort included 8,562 patients (Supplementary Material Table S2). There are no significant differences for the baseline characteristics between the two cohorts (*P* > 0.05).

**Figure 1:**
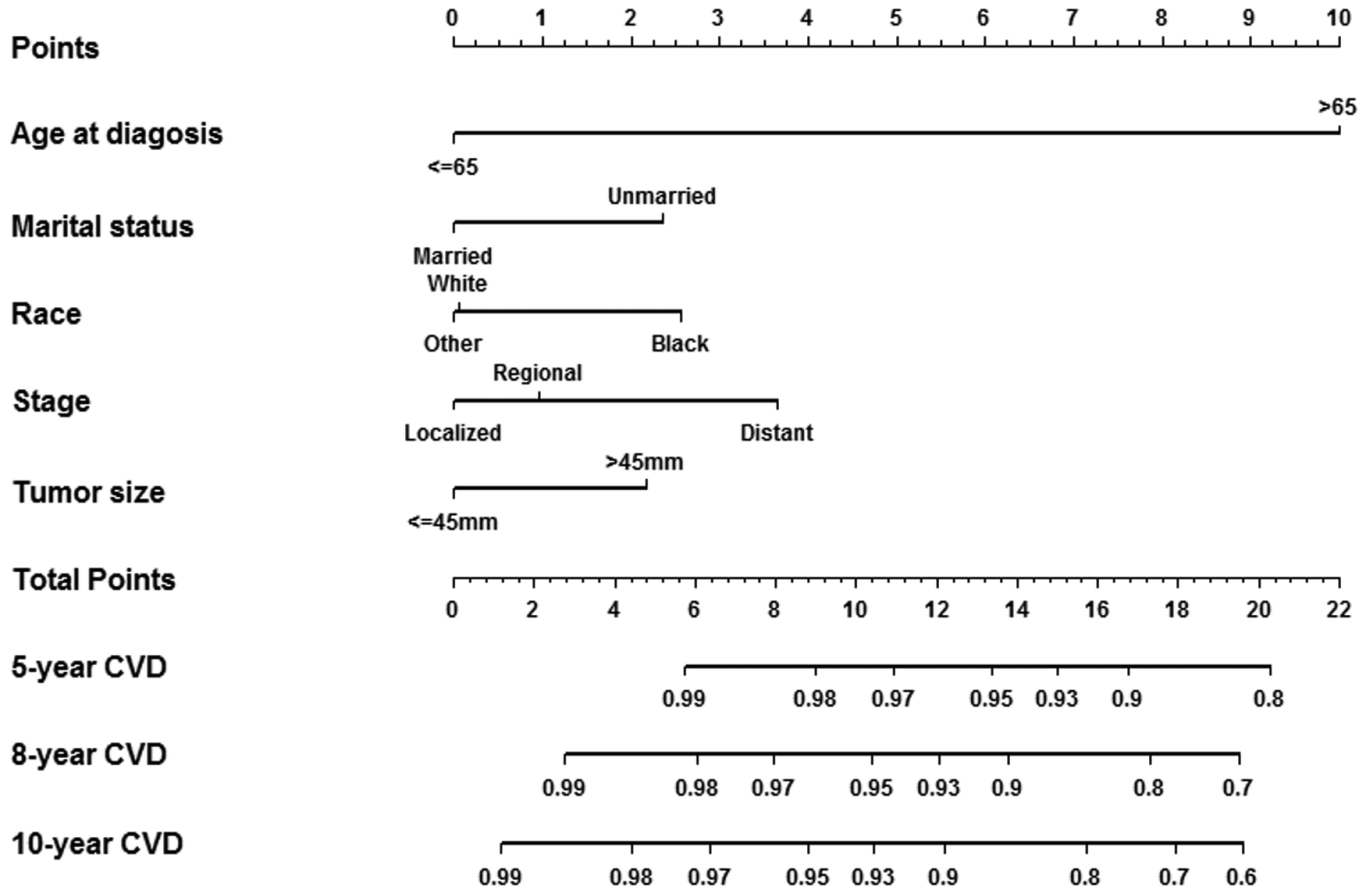
Nomogram predicting survival in breast cancer patients with CT or RT.

In univariate and multivariate analyses for training cohort, tumor size, stage, race and marital status were related to the risk of CVD death (Supplementary Material Table S3). According to the results, a nomogram was generated to predict the 5-year, 8-year, and 10-year risk of CVD survival in breast cancer patients with CT or RT. As shown in Figure 1 and Supplementary Material Table S4, age at diagnosis was assigned a maximum score of 10, followed by stage, race, marital status, and tumor size, respectively. The aggregate score obtained by summing the scores of the five variables corresponds to the risk of CVD survival in the next 5, 8, and 10 years. For example, for a patient in the database, 70 years old had a score of 10.0, married had a score of 0, 60 mm tumor size was 2.1, black had a score of 2.6, and a localized stage had a score of 0, and a total score was 14.7. The risk of CVD survival in the next 5 years was 93% to 95%, the risk of CVD survival in 8 years was 85% to 87%, and the risk of CVD survival in 10 years was 82% to 85%.

**Figure 2:**
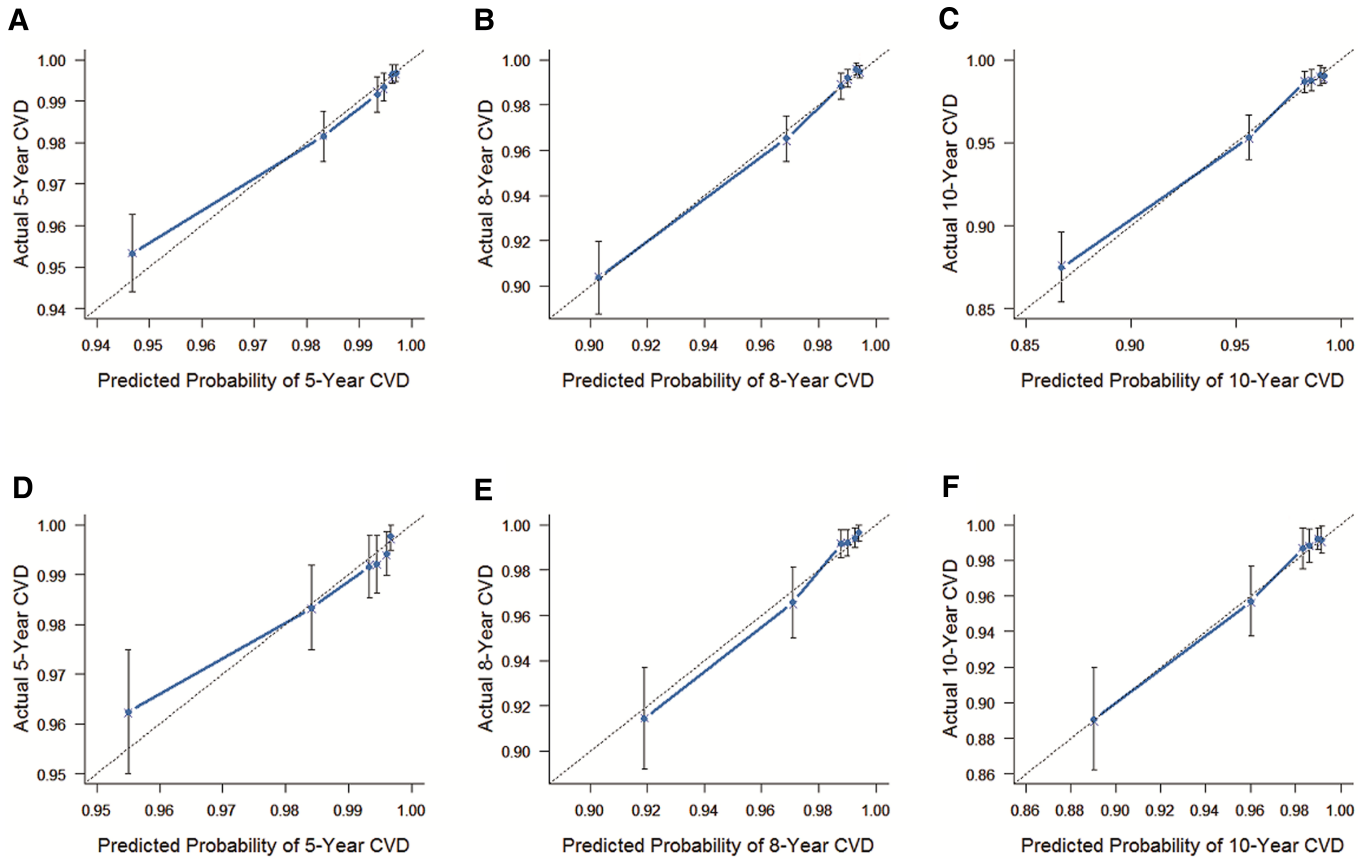
The C-index of the internal and external validation. (**A**) The internal validation cohort was validated within 5 years. (**B**) The internal validation cohort was validated within 8 years. (**C**) The internal validation cohort was verified within 10 years. (**D**) The external validation cohort was validated within 5 years. (**E**) The external validation cohort was validated within 8 years. (**F**) The external validation cohort was validated within 10 years. The 45° dashed line represents a perfect match between the actual survival outcome (Y-axis) and the nomogram predicted survival outcome (X-axis). The more the blue dashed line fits the 45° dashed line, the more accurate the model is.

The C-index of the internal validation was 0.780 (95% Cl = 0.751–0.809), and that of external validation was 0.809 (95% Cl = 0.768–0.850), reflecting the high accuracy of the model. As shown in Figure 2, results of the calibration curves based on internal validation in the training cohort and external validation in the validation cohort showed that the 5-year, 8-year, and 10-year CVD survival prediction rates were close to the actual risk proportion of CVD survival.

### 3.4. Risk stratification for CVD death

**Figure 3:**
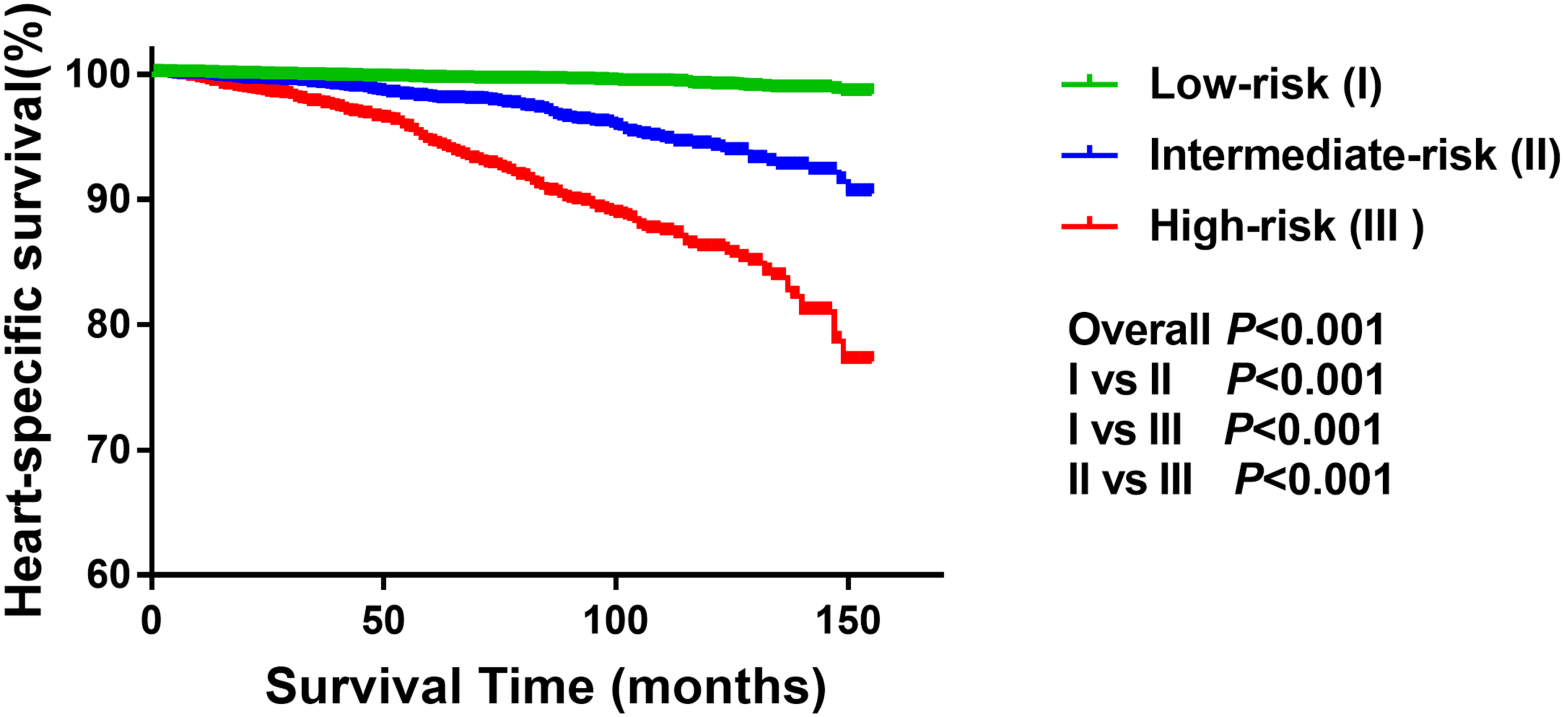
Three risk groups of survival in breast cancer patients with CT or RT.

The risk stratification of CVD death in breast cancer patients with CT or RT was constructed based on the overall score predicted by nomogram, and divided into low-risk group, intermediate-risk group and high-risk group. As is shown in Figure 3, the risk of CVD death in the low-risk group was lower than that in the intermediate-risk group and the high-risk group, and the risk of CVD death in the high-risk group was the highest. The *P* value for pairwise comparisons within groups <0.001 suggested that this risk stratification can accurately reflect the CVD death in breast cancer patients with CT or RT.

## 4. Discussion

In this population-based study, we for the first time assessed the impact of tumor characteristics on CVD death risk in breast cancer patients with CT or RT and found that tumor size and stage were related to the risk of CVD death for breast cancer patients with CT or RT.

Previous studies on the cardio-toxicity of CT and RT have been comprehensive and have reached consistent conclusions (4–8). Many chemotherapeutic drugs, such as anthracyclines, could lead to a production of reactive oxygen species and form the complex of anthracyclines and iron, producing toxic hydroxyl and radicals that with cardiotoxicity (12). As for after radiotherapy, the release of inflammatory cytokines and reactive oxygen species increases, promoting radiation fibrosis and causing direct damage to the DNA and vascular endothelium, thereby promoting the occurrence and development of CVD (30–32). Since the characteristic of high risk of CVD death, we focus on those breast patients who accepted CT or RT to identify risk factors of CVD death, especially the tumor characteristics.

The association between tumor size and prognosis is well recognized (33–40). However, most previous studies focused on the relationship between tumor size and overall survival (OS) and cancer-specific survival (CSS) in breast cancer, ignoring its effect on CVD death (33–35). We found that for breast cancer patients with CT or RT, those with size ≥ 45 mm breast cancer had higher risk of CVD death than those with size <45 mm breast cancer. Our results are similar to the findings of Leoce NM et al*.* who subdivided tumor size into ≤2 cm, 2–5 cm and >5 cm and reported a higher risk of CVD for tumor measuring > 5 cm among patients diagnosed with stage I—III breast cancer (36). However, they did not exclude the interference of missing or unknown cases, and the critical value of the tumor size was targeted for OS but not for CVD, making the results less reliable. In our study, we stratified the tumor size using X-tile software based on the minimum *P* value and maximum *χ*^2^ (22). The optimal cut-off value of tumor size was a powerful and objective predictor of CVD death in breast cancer patients with CT or RT. As previously described in researches, the dose and intensity of anticancer therapy likely increase with tumor size (37–39), which may subsequently raise the risk of CVD death in breast cancer patients with CT or RT because of the cardio-toxicity (40). Therefore, patients with breast cancer measuring ≥45 mm require additional cardiovascular monitoring and care.

According to the previous studies, tumor stage has been identified as one of prognostic factors for breast cancer patients with CT or RT, while its effect on CVD death has received less attention (41, 42). Our study found that breast cancer patients with CT or RT with regional and distant stage were more strongly associated with CVD death risk than those with localized stages. Stoltzfus KC et al. reported that cancer patients with distant stage had the highest standardized motality ratio of death from fatal heart disease (43). Considering the heterogeneity among different cancers, the death risk of fatal heart disease among overall cancer survivors is not representative of breast cancer specifically. Existing studies have also indicated that cardiotoxicity of RT and CT are particularly important for patients with metastatic disease, in whom the intensity of anticancer treatment is high, predisposing patients to CVD events (39, 44, 45). Furthermore, when breast cancer develops with cachexia, it can lead to cardiometabolic disorders such as cardiac fibrosis and cardiac atrophy (46), increasing the risk of CVD death.

Tumor size and stage, characteristics of breast cancer, reflect the influence of breast cancer itself on CVD. Increasing clinical and basic investigations found that breast cancer itself may lead to cardiovascular complications (13, 15–17). A new breast cancer diagnosis is related to an increased risk of CVD death independently (14). Breast cancer leads to CVD by inducting the pro-inflammatory effects and cytotoxicity of neutrophil extracellular traps (NETs) (16, 17). Furthermore, tumor cells lead to hypercoagulability of blood in three parts: endothelial injury, increased coagulability, and inhibited fibrinolysis (17, 47, 48). All these observations support our result and suggest that the influence of breast tumor characteristics on the risk of CVD death needs further investigation by oncology and cardiovascular physicians.

The effect of tumour laterality on the risk of CVD death in breast cancer with RT remains controversial. Similarly, numerous studies indicated no excess of cardiac diseases and mortality among breast patients received left-sided radiotherapy compared with the right-sided group (49–51). Our study did not observe a statistically significant association of tumor laterality with CVD death. The effect of tumour laterality on the risk of CVD death in breast cancer with RT needed to be further explored.

Most previous prediction models of CVD death risk, including demographic variables (such as age, race, etc.) or the risk factors of CVD (such as smoking, hypertension, etc.), were little considered tumor features as predictors (52–54). Variability among different tumor characteristics reminds us that prediction models of CVD death can be more comprehensive and personalized by including risk factors synthetically. Additionally, incomplete consideration of the potential influence of tumor characteristics on CVD death risk may be biased or deteriorated, and breast cancer patients with CT or RT may lose optimal clinical management. Our result complements the deficiencies of existing prediction models. In addition, although our predictive nomogram still needs to be improved, the model can visualize the impact of tumor characteristics on the risk of CVD death in a simple graph. By incorporating tumor characteristics and other significant clinical factors, the model can help to individuate risk assessments and clinical preventive strategies for CVD and supply the limitations of the TNM staging (55). Therefore, further nomograms for predicting CVD death risk in breast cancer patients with CT or RT should incorporate tumor characteristics to improve accuracy and predictive value.

With the emerging field of cardio-oncology, cardiovascular care has become an important consideration for cancer patients (15). According to the American Heart Association management and clinical practice guideline, CVD risk monitoring should be performed in cancer patients who have received cardio-toxicity treatment (such as radiotherapy, chemotherapy, targeted therapy, etc.) (6, 56–58). However, the impact of tumor characteristics on prevention strategies for CVD death risk cannot be ignored. Our study found that breast cancer patients with CT or RT may have different assessment of CVD death risk with the influence of tumor size and stage. Therefore, according to the impact of tumor characteristics, improving management strategies for the risk of CVD death can help improve the quality of care and prognosis for breast cancer patients with CT or RT.

## 5. Strengths and limitations

The remarkable strengths of our study were the long follow-up time and large multicenter sample size. To our knowledge, our study is one of the largest and first studies evaluating the impact of tumor characteristics on the risk of CVD death among breast cancer patients with CT or RT.

Our study has some limitations. Firstly, similar to previous studies (8, 13, 23, 59), the SEER database does not provide detailed data on the CT or RT, cardiovascular comorbidities and risk factors, and we could not further explore their impact on the risk of CVD death. A particular focus of further studies should be the stratification analyses of the dose and type of both CT or RT to reveal the potential effect of tumor size and stage on CVD death risk. Secondly, information on hormone therapy does not provide by the SEER database. Thirdly, the nomogram of tumor size and stage on CVD death should be further verified in multicenter validation cohort. It should be noted that the nomogram was mainly used to visualize the impact of tumor characteristics on the risk of CVD death and constructing predicted model was not the primary aim in our study.

## 6. Conclusions

Tumor size > 45 mm, regional and distant stage were risk factors of CVD death among breast cancer patients with CT or RT. The management of CVD death risk in breast cancer patients with CT or RT should focus not only on the risk factors of CVD but also on tumor characteristics, especially tumor size and stage. These findings may offer new insights and population-based scientific basis for decreasing CVD death risk and management of breast cancer patients with CT or RT.

## Data Availability

The datasets presented in this study can be found in online repositories. The names of the repository/repositories and accession number(s) can be found below: http://seer.cancer.gov.
