# Supplementary material for: The impact of tumor characteristics on cardiovascular disease death in breast cancer patients with CT or RT: a population-based study
Source: Front Cardiovasc Med. 2023 May 9;10:1149633. doi: 10.3389/fcvm.2023.1149633 (PMC10203988; doi:10.3389/fcvm.2023.1149633)
Supplement: Supplementary file 1 [file Table1.docx]

***Supplementary Material***

**The impact of tumor characteristics on cardiovascular**

**disease death in breast cancer patients with CT or RT: A**

**population-based study**

**Kaiyi Chi , Zehao Luo, Hongjun Zhao , Yemin Li, Yinglan Liang ,**

**Zhaoling Xiao, Yiru He , Hanbin Zhang , Zaiying Ma, Liangjia Zeng ,**

**Ruoyun Zhou , Manting Feng, Wangen Li,Huying Rao*, Min Yi***

***Corresponding author:** Min Yi: smu_min@126.com;

Huying Rao: 542083902@qq.com.

**List of Supplementary Tables**

Supplementary Table S1. Univariate analysis of CVD death risk in 28,539 breast cancer patients with CT or RT

Abbreviations: CVD, Cardiovascular Disease; CT, chemotherapy; RT, radiotherapy; HR, hazard ratio; CI, confidence interval; ER, estrogen receptor; PR, progesterone receptor; HER2, human epidermal receptor 2.

Supplementary Table S2. Baseline characteristics of training cohort and validation cohort

Abbreviations: ER, estrogen receptor; PR, progesterone receptor; HER2, human epidermal receptor 2.

Supplementary Table S3. Analysis of univariate and multivariate analysis in training cohort

Abbreviations: ER, estrogen receptor; PR, progesterone receptor; HER2, human epidermal receptor 2; NI, not included.

Supplementary Table S4. Specific scores of prognostic factors in the nomogram model

**List of Supplementary Figures**

Supplementary Figure S1. X-tile analysis of survival data based on tumor size

Supplementary Figure S2. X-tile analysis of survival data based on nomogram score of each patient

**Supplementary Methods**

**Histological subtype:**

**1.ductal:** 8500/3, 8501/3, 8503/3, 8507/3, 8521/3

**2.Lobular:** 8520/3

**3.mixed:** 8522/3,8523/3,8524/3,8541/3,8543/3

**4.other:** 8000/3,8001/3,8004/3,8010/3,8012/3,8013/3,8020/3,8021/3,8022/3,8032/3,8033/3,8035/3,8041/3,8045/3,8046/3,8050/3,8052/3,8070/3,8071/3,8074/3,8090/3,8123/3,8140/3,8141/3,8200/3,8201/3,8211/3,8230/3,8240/3,8246/3,8251/3,8255/3,8260/3,8310/3,8315/3,8323/3,8341/3,8343/3,8401/3,8403/3,8413/3,8430/3,8440/3,8450/3,8480/3,8481/3,8490/3,8502/3,8504/3,8510/3,8512/3,8513/3,8525/3,8530/3,8540/3,8542/3,8550/,8560/3,8570/3,8571/3:,8572/3,8573/3,8574/3,8575/3,8720/3,8800/3,8801/3,8802/3,8805/3,8810/3,8811/3,8830/3,8832/3,8836/3,8850/3,8851/3,8854/3,8858/3,8890/3,8895/3,8900/3,8920/3,8935/3,8980/3,8982/3,8983/3,9020/3,9041/3,9044/3,9120/3,9180/3,9580/3,9590/3,9591/3,9650/3,9652/3,9663/3,9670/3,9671/3,9673/3,9680/3,9684/3,9687/3,9690/3,9691/3,9695/3,9698/3,9699/3,9702/3,9714/3,9728/3,9731/3,9734/3,9750/3,9823/3,9837/3,9930/3

**Supplementary Table S1.** Univariate analysis of CVD death risk in 28,539 breast cancer patients with CT or RT

| Variables | **HR (95% CI)** | ***P* Value** |
| --- | --- | --- |
| **Age at diagnosis** |  | <0.001 |
| ≤65 | Reference |  |
| >65 | 9.109(7.454-11.132) | <0.001 |
| **Race** |  | <0.001 |
| White | Reference |  |
| Black | 1.728(1.356-2.202) | <0.001 |
| Other* | 0.744(0.503-1.101) | 0.140 |
| **Marital status** |  | <0.001 |
| Married | Reference |  |
| Unmarried | 2.547(2.112-3.071) | <0.001 |
| **Laterality** |  | 0.114 |
| Right | Reference |  |
| Left | 1.161(0.965-1.398) | 0.114 |
| **Histologic subtypes** |  | 0.565 |
| Ductal | Reference |  |
| Lobular | 1.200(0.876-1.644) | 0.256 |
| Mixed | 0.997(0.754-1.319) | 0.986 |
| Other | 1.225(0.796-1.887) | 0.356 |
| **Tumor Size** |  | <0.001 |
| ≤45mm | Reference |  |
| >45mm | 1.551(1.229-1.957) | <0.001 |
| **Year of diagnosis** |  | 0.171 |
| 2004-2007 | Reference |  |
| 2008-2011 | 0.991(0.798-1.232) | 0.938 |
| 2012-2016 | 0.718(0.501-1.028) | 0.071 |
| **Grade** |  | 0.321 |
| Low | Reference |  |
| High | 1.099(0.912-1.325) | 0.321 |
| **Stage** |  | <0.001 |
| Localized | Reference |  |
| Regional | 1.143(0.945-1.383) | 0.168 |
| Distant | 2.391(1.572-3.636) | <0.001 |
| **ER status** |  | 0.966 |
| Negative | Reference |  |
| Positive | 0.995(0.792-1.250) | 0.966 |
| **PR status** |  | 0.643 |
| Negative | Reference |  |
| Positive | 0.954(0.783-1.163) | 0.643 |
| **HER2 status** |  | 0.020 |
| Negative | Reference |  |
| Positive | 0.798(0.464-1.371) | 0.413 |
| Unknown | 1.356(1.035-1.777) | 0.027 |
| **Surgery** |  | 0.175 |
| No evidence | Reference |  |
| Yes | 0.595(0.281-1.260) | 0.175 |

*Other includes American Indian/Alaska Native and Asian/Pacific Islander.

Abbreviations: CVD, Cardiovascular Disease; CT, chemotherapy; RT, radiotherapy; HR, hazard ratio; CI, confidence interval; ER, estrogen receptor; PR, progesterone receptor; HER2, human epidermal receptor 2.

**Supplementary Table** S2. Baseline characteristics of training cohort and validation cohort

| Variables | **Training cohort（n=19,977）** | | **Validation cohort (n=8,562)** | | ***P* Value** |
| --- | --- | --- | --- | --- | --- |
|  | **n** | **%** | **n** | **%** |  |
| **Age at diagnosis** |  |  |  |  | 0.974 |
| ≤65 | 15,517 | 77.7 | 6,649 | 77.7 |  |
| >65 | 4,460 | 22.3 | 1,913 | 22.3 |  |
| **Race** |  |  |  |  | 0.439 |
| White | 15,680 | 78.5 | 6,696 | 78.2 |  |
| Black | 2,491 | 12.5 | 1,052 | 12.3 |  |
| Other* | 1,806 | 9.0 | 814 | 9.5 |  |
| **Marital status** |  |  |  |  | 0.803 |
| Married | 12,416 | 62.2 | 5,308 | 62.0 |  |
| Unmarried | 7,561 | 37.8 | 3,254 | 38.0 |  |
| **Laterality** |  |  |  |  | 0.494 |
| Right | 9,737 | 48.7 | 4,211 | 49.2 |  |
| Left | 10,240 | 51.3 | 4,351 | 50.8 |  |
| **Histologic subtypes** |  |  |  |  | 0.113 |
| Ductal | 15,075 | 75.5 | 6,372 | 74.4 |  |
| Lobular | 1,769 | 8.9 | 750 | 8.8 |  |
| Mixed | 2,367 | 11.8 | 1,097 | 12.8 |  |
| Other | 766 | 3.8 | 343 | 4.0 |  |
| **Tumor Size** |  |  |  |  | 0.262 |
| ≤45mm | 16,319 | 81.7 | 7,042 | 82.2 |  |
| >45mm | 3,658 | 18.3 | 1,520 | 17.8 |  |
| **Year of diagnosis** |  |  |  |  | 0.615 |
| 2004-2007 | 5,845 | 29.3 | 2,456 | 28.7 |  |
| 2008-2011 | 6,333 | 31.7 | 2,742 | 32.0 |  |
| 2012-2016 | 7,799 | 39.0 | 3,364 | 39.3 |  |
| **Grade** |  |  |  |  | 0.722 |
| Low | 11,448 | 57.3 | 4,926 | 57.5 |  |
| High | 8,529 | 42.7 | 3,636 | 42.5 |  |
| **Stage** |  |  |  |  | 0.608 |
| Localized | 8,636 | 43.2 | 3,718 | 43.4 |  |
| Regional | 10,240 | 51.3 | 4,397 | 51.4 |  |
| Distant | 1,101 | 5.5 | 447 | 5.2 |  |
| **ER status** |  |  |  |  | 0.549 |
| Negative | 4,331 | 21.7 | 1,829 | 21.4 |  |
| Positive | 15,646 | 78.3 | 6,733 | 78.6 |  |
| **PR status** |  |  |  |  | 0.974 |
| Negative | 6,473 | 32.4 | 2,776 | 32.4 |  |
| Positive | 13,504 | 67.6 | 5,786 | 67.6 |  |
| **HER2 status** |  |  |  |  | 0.439 |
| Negative | 8,170 | 40.9 | 3,536 | 41.3 |  |
| Positive | 2,398 | 12.0 | 1,059 | 12.4 |  |
| Unknown | 9,409 | 47.1 | 3,967 | 46.3 |  |
| **Surgery** |  |  |  |  | 0.136 |
| No evidence | 521 | 2.6 | 250 | 2.9 |  |
| Yes | 19,456 | 97.4 | 8,312 | 97.1 |  |

*Other includes American Indian/Alaska Native and Asian/Pacific Islander.

Abbreviations: ER, estrogen receptor; PR, progesterone receptor; HER2, human epidermal receptor 2.

**Supplementary** Table S3. Analysis of univariate and multivariate analysis in training cohort

| Variables | **Univariate analysis** | | **Multivariate analysis** | |
| --- | --- | --- | --- | --- |
|  | **HR (95% CI)** | ***P* Value** | **HR (95% CI)** | ***P* Value** |
| **Age at diagnosis** |  | <0.001 |  | <0.001 |
| ≤65 | Reference |  | Reference |  |
| >65 | 8.476(6.699-10.726) | <0.001 | 8.409(6.615-10.691) | <0.001 |
| **Race** |  | <0.001 |  | 0.001 |
| White | Reference |  | Reference |  |
| Black | 1.792(1.348-2.383) | <0.001 | 1.702(1.273-2.276) | <0.001 |
| Other* | 0.800(0.507-1.262) | 0.336 | 0.988(0.625-1.560) | 0.957 |
| **Marital status** |  | <0.001 |  | <0.001 |
| Married | Reference |  | Reference |  |
| Unmarried | 2.425(1.943-3.026) | <0.001 | 1.653(1.315-2.077) | <0.001 |
| **Laterality** |  | 0.257 | NI |  |
| Right | Reference |  |  |  |
| Left | 1.136(0.911-1.415) | 0.257 |  |  |
| **Histologic subtypes** |  | 0.326 | NI |  |
| Ductal | Reference |  |  |  |
| Lobular | 1.257(0.868-1.820) | 0.226 |  |  |
| Mixed | 1.068(0.768-1.484) | 0.696 |  |  |
| Other | 1.450(0.897-2.343) | 0.129 |  |  |
| **Tumor Size** |  | <0.001 |  | 0.002 |
| ≤45mm | Reference |  | Reference |  |
| >45mm | 1.630(1.244-2.135) | <0.001 | 1.591(1.192-2.123) | 0.002 |
| **Year of diagnosis** |  | 0.118 | NI |  |
| 2004-2007 | Reference |  |  |  |
| 2008-2011 | 1.008(0.780-1.301) | 0.953 |  |  |
| 2012-2016 | 0.649(0.420-1.003) | 0.051 |  |  |
| **Grade** |  | 0.156 | NI |  |
| Low | Reference |  |  |  |
| High | 1.173(0.941-1.463) | 0.156 |  |  |
| **Stage** |  | 0.003 |  | 0.009 |
| Localized | Reference |  | Reference |  |
| Regional | 1.110(0.886-1.392) | 0.365 | 1.225(0.966-1.552) | 0.094 |
| Distant | 2.348(1.433-3.849) | 0.001 | 2.173(1.297-3.641) | 0.003 |
| **ER status** |  | 0.876 | NI |  |
| Negative | Reference |  |  |  |
| Positive | 1.022(0.777-1.344) | 0.876 |  |  |
| **PR status** |  | 0.981 | NI |  |
| Negative | Reference |  |  |  |
| Positive | 0.997(0.787-1.263) | 0.981 |  |  |
| **HER2 status** |  | 0.001 | NI |  |
| Negative | Reference |  |  |  |
| Positive | 0.918(0.473-1.780) | 0.800 |  |  |
| Unknown | 1.751(1.245-2.463) | 0.001 |  |  |
| **Surgery** |  | 0.187 | NI |  |
| No evidence | Reference |  |  |  |
| Yes | 0.550(0.227-1.337) | 0.187 |  |  |

*Other includes American Indian/Alaska Native and Asian/Pacific Islander.

Abbreviations: ER, estrogen receptor; PR, progesterone receptor; HER2, human epidermal receptor 2; NI, not included.

**Supplementary Table S4.** Specific scores of prognostic factors in the nomogram model

| **Variables** | **Nomogram Point** |
| --- | --- |
| **Age at diagnosis** |  |
| ≤65 | 0.0 |
| >65 | 10.0 |
| **Marital status** |  |
| Married | 0.0 |
| Unmarried | 2.4 |
| **Race** |  |
| Other* | 0.0 |
| White | 0.1 |
| Black | 2.6 |
| **Stage** |  |
| Localized | 0.0 |
| Regional | 1.0 |
| Distant | 3.6 |
| **Tumor Size** |  |
| ≤45mm | 0.0 |
| >45mm | 2.1 |

*Other includes American Indian/Alaska Native and Asian/Pacific Islander.


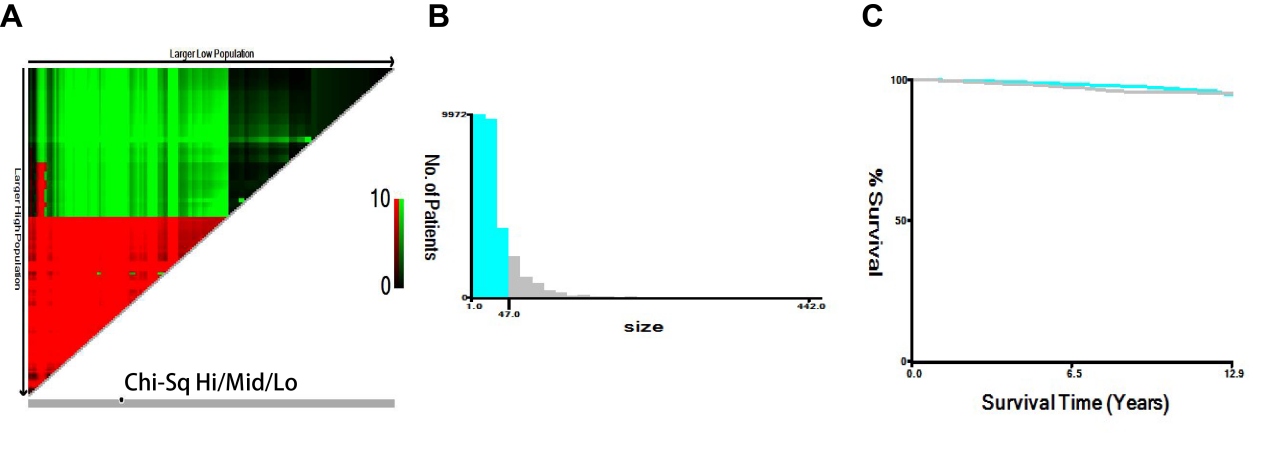


**Supplementary Figure S1.** X-tile analysis of survival data based on tumor size.

Picture (A) shows that the optimal cut-point of tumor size was defined as the brightest pixel and 47 mm was identified as the optimal cutoff point by X-tile determination (*P*=0.0026). The result of picture (B) Histogram and picture (C) Kaplan Meier's analysis were calculated using the above cutoff value.


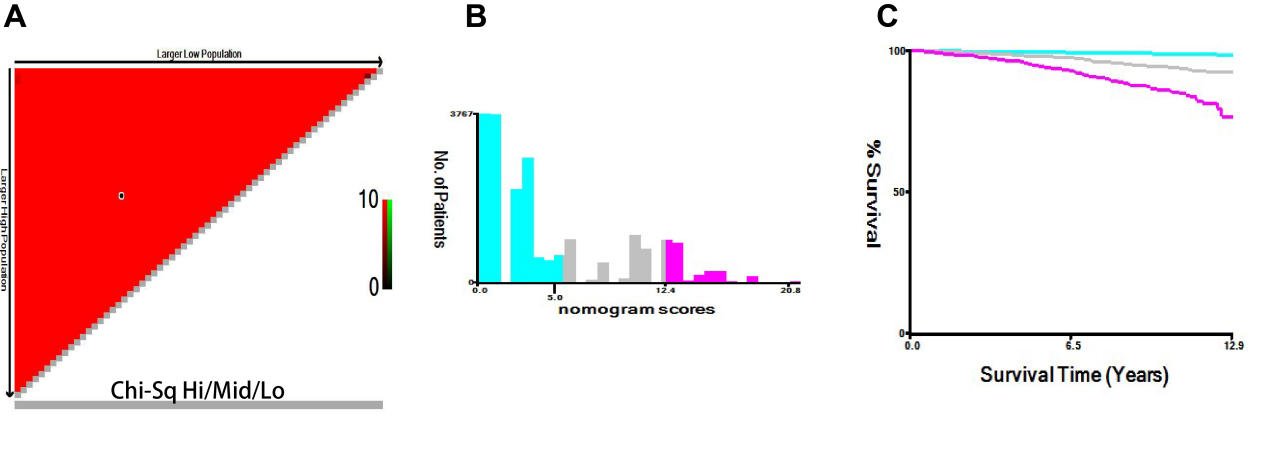


**Supplementary Figure S2.** X-tile analysis of survival data based on nomogram score of each patient. Picture (A) shows that 5 and 12.4 scores were identified as the best cutoff point by X-tile determination(*P*<0.01). The result of picture (B) Histogram and picture (C) Kaplan Meier's analysis were calculated using the above cutoff value.
